# Lusus Naturæ

**Published:** 1886-06

**Authors:** R. S. Gregg

**Affiliations:** Manor, Texas


### LUSUS NATURÆ.

MANOR, TEXAS, February 22, 1886.

EDIIOR DANIEL'S MEDICAL JOURNAL:

On the night of the 2nd day of this month, I was called to attend Mrs. B. in her second confinement. About fifteen minutes after my arrival, was informed that the "bag of waters" had ruptured, and that this was the first indication of approaching labor. I immediately made a digital examination, and found, to my surprise, the vagina filled with a soft mass, that felt more like the small intestines, than anything else that I could imagine. Upon further examination, I found that the entire mass projected from the os uteri, which was dilated to the size of a silver dollar. I then came to the conclusion that I had a prolapsed cord, and also shoulder presentation to deal with, and finding no pulsation in what I had decided was an abnormal cord, I informed the husband of the lady, that I thought the child was dead; and as the os was still too small to introduce my hand to perform version, I feared trouble in the delivery. I then asked for consultation, and he sent a messenger for my friend, Dr. W. T. Richmond, to come to my assistance. When the Doctor arrived, he made a digital examination, and very honestly said that he could not be certain what was presenting, but that it felt to him more like intestines than anything else, yet it might be an abnormal cord. The Doctor then kindly administered chloroform for me, and upon further examination I found the child small, and had very little difficulty in pulling down both feet and delivering. We had before us what we considered a real curiosity; and as the husband refused to let us have the fœtus, I will endeavor to give you a description of it. The most concise description would be to say that we had before us a case of *spontaneous evisceration*. The mother said that she had not expected her confinement until March 1st, yet the fœtus appeared mature; head was perfect, left arm absent, left ribs except last two, absent, sternum absent, thoracic and abdominal walls absent on left side, beginning at a point where the left clavicle should have been attached to sternum, and extending downward along the median line a little below the umbilicus, thence to a point near junction of the last lumbar and first sacral vertibrae, thence upward along the spinal column to the lower portion of the scapula, thence obliquely to the sternal notch. The spinal column was very much curved toward the left side. The heart and a rudimentary left lung were protruding from the extreme upper angle of this opening; just below them the liver, and what appeared to be an enlarged stomach; and on left side of the opening was an enlarged kidney with a broken ureter hanging from it. Below this were the large and small intestines, these having constituted the mass that so puzzled Dr. R. and myself before delivery of the fœtus. At the extreme lower angle of the aperture was the bladder distended with urine and attached to it the other end of the broken ureter. The mother and father of this freak of nature, are both healthy and well developed persons. The father about 24 or 25 years of age and the mother 23 years. During the early months of this pregnancy, the mother was troubled with chills and fever, and last fall she had dengue, but at the time of this confinement her health was good. I forgot to say that the child from the first pregnancy was well developed, but died last summer from dysentery, complicating dentition. So far as I was able to learn there was no fright or nervous shock of any kind to apparently account for such an unfortunate deformity in the fœtus. Hoping that this report may be of some interest to you and the readers of your sprightly journal, I remain,

Yours Fraternally,

R. S. Gregg.

